# An Algorithm for the Selection of Probes for Specific Detection of Human Disease Pathogens Using the DNA Microarray Technology

**DOI:** 10.17691/stm2022.14.1.01

**Published:** 2022-01-28

**Authors:** E.N. Filatova, A.S. Chaikina, N.F. Brusnigina, M.A. Makhova, O.V. Utkin

**Affiliations:** Leading Researcher, Laboratory of Molecular Biology and Biotechnology; Blokhina Scientific Research Institute of Epidemiology and Microbiology of Nizhny Novgorod, Federal Service for Surveillance on Consumer Rights Protection and Human Wellbeing (Rospotrebnadzor), 71 Malaya Yamskaya St., Nizhny Novgorod, 603950, Russia;; Student; Privolzhsky Research Medical University, 10/1 Minin and Pozharsky Square, Nizhny Novgorod, 603005, Russia; Associate Professor, Head of the Laboratory for Metagenomics and Molecular Indication of Pathogens; Blokhina Scientific Research Institute of Epidemiology and Microbiology of Nizhny Novgorod, Federal Service for Surveillance on Consumer Rights Protection and Human Wellbeing (Rospotrebnadzor), 71 Malaya Yamskaya St., Nizhny Novgorod, 603950, Russia;; Senior Researcher, Laboratory for Metagenomics and Molecular Indication of Pathogens; Blokhina Scientific Research Institute of Epidemiology and Microbiology of Nizhny Novgorod, Federal Service for Surveillance on Consumer Rights Protection and Human Wellbeing (Rospotrebnadzor), 71 Malaya Yamskaya St., Nizhny Novgorod, 603950, Russia;; Head of the Laboratory of Molecular Biology and Biotechnology; Blokhina Scientific Research Institute of Epidemiology and Microbiology of Nizhny Novgorod, Federal Service for Surveillance on Consumer Rights Protection and Human Wellbeing (Rospotrebnadzor), 71 Malaya Yamskaya St., Nizhny Novgorod, 603950, Russia;

**Keywords:** probe selection algorithm, DNA microarray, DNA microarray design, community-acquired pneumonia, Chlamydophila pneumoniae

## Abstract

**The aim of the study** was to develop an algorithm for the selection of discriminating probes to identify a wide range of causative agents of human infectious diseases.

**Materials and Methods:**

The algorithm for selecting the probes was implemented in the form of the disprose (DIScrimination PRObe SElection) computer program written in the R language. Additionally, third-party software was used: the BLAST+ and ViennaRNA Package programs. The developed algorithm was tested by selecting specific probes for detecting *Chlamydophila (Chlamydia) pneumoniae* — an atypical bacterial pathogen causing community-acquired pneumonia (CAP). Nucleotide sequences for analysis were downloaded from the NCBI databank.

**Results:**

An algorithm for the selection of specific probes capable of detecting human infectious pathogens has been developed. The algorithm is implemented in the form of the disprose modular program, which allows for performing all stages of the probe selection process: loading the nucleotide sequences and their metadata from available databanks, creating local databases, forming a pool of probes, calculating their physicochemical parameters, aligning the probes and sequences contained in local databases, processing and evaluating the alignment results. The algorithm was successfully tested and its performance was confirmed by selecting a set of probes for the specific detection of *Chlamydophila pneumoniae*. The specificity of the selected probes calculated *in silico* indicated a low risk of their nonspecific binding and a high potential of using them as molecular genetic diagnostic tools (DNA microarrays, PCR).

**Conclusion:**

An algorithm for the selection of specific probes detecting a wide range of human pathogens in clinical biomaterial has been developed and implemented in the form of the disprose modular program. The probes selected using this program can serve as the functional basis of DNA-oriented microarrays able to identify causative agents of polyetiological diseases, such as CAP. Due to the flexibility and openness of the program, the scope of its application can be expanded.

## Introduction

In view of the current epidemic situation, the need for diagnostic DNA microarrays capable of detecting bacterial and viral pathogens has become urgent.

The diagnostic value of a DNA microarray depends on the selection of specific oligonucleotide probes. There are difficulties with this selection, which are due to the chemical complexity and species specificity of biological samples; these factors contribute to the high risk of probe cross-hybridization with a non-target DNA, and the appearance of false positive results [1].

The existing algorithms for the probe selection are based on assessing the conformity of candidate sequences to the criteria of specificity and homogeneity [2–5]. As a rule, the software that implements the known algorithms does not allow the user to modify either these criteria or the order of their application and/or parameters. However, the weight of the criteria and their appropriateness may vary depending on the spectrum of detected pathogens, their taxonomic diversity, the biological sample specifics, and the purpose of the examination (identification of pathogenic factors, determination of antibiotic resistance, etc.). Any additional complication in the procedure for probe selection inevitably leads to an increase in the time and labor, as well as an increase in the requirements for computing equipment. In this regard, we suggest that algorithms implemented in the form of modular/modifiable programs are preferable for the selection of specific probes to be used in diagnostic DNA microarrays.

**The aim of the study** was to develop an algorithm for the selection of discriminating probes to identify a wide range of causative agents of human infectious diseases.

## Materials and Methods

In this study, we pursued the following specific goals: to develop an algorithm for the selection of specific probes, to implement it in the form of a computer program, to optimize the performance of the algorithm, and to test it by selecting probes for the detection of *Chlamydophila (Chlamydia) pneumoniae.*

The algorithm for the selection of probes intended for the mass differential detection of bacterial and viral pathogens is implemented in the form of the disprose computer program written in the R programming language and designed as a package of functions. The package is distributed under the GNU GPL-3 license (2007) and is available for downloading from The Comprehensive R Archive Network official international repository (https://CRAN.R-project.org/package=disprose).

The computations were performed using an Intel Xeon 2560 (x2) workstation, 128 GB RAM. The algorithm was tested by searching for probes that allow specific detection of the “atypical” causative agent of community-acquired pneumonia (CAP) — *C. pneumoniae*. The genetic sequences of *C. pneumoniae* were obtained from the NCBI Nucleotide database [6].

To calculate the minimal folding energy (MFE) of the oligonucleotide sequences in the candidate probes, we used the ViennaRNA Package (version 2.4.14) [7, 8]. The melting point (Tm) was calculated based on the established set of thermodynamic parameters [9] by the nearest neighbor method [10].

Local alignment of nucleotide sequences was carried out using the blastn program from the BLAST+ program package (version 2.10.0) [11]. The search for matches was performed in the full-size downloadable NCBI Nucleotide collection database, as well as in local databases of nucleotide sequences generated using the blastdbcmd program.

This work did not use information that might violate anyone’s confidentiality. No human or animal subjects were involved in the study.

## Results

### Algorithm for the selection of discriminating probes and its implementation in the disprose program

#### Selection of target nucleotide sequences

Before starting the procedure for selecting the probes, it was necessary to discern between the target nucleotide sequences (to make the probes hybridize) and nonspecific sequences (to avoid hybridization).

The target sequences can be obtained by the researchers themselves or downloaded from the available databanks. To date, the disprose program implements the functions of downloading the sequences and their metadata from several large banks: NCBI (Nucleotide, GenBank, RefSeq databases) and GISAID. Based on the metadata obtained, the researcher can select the sequences of interest from the entire set of available data. The selected nucleotide sequences constitute a local base of target sequences for testing the ability of candidate probes to hybridize to these sequences.

#### Selection of nonspecific sequences

To date, the downloadable NCBI Nucleotide collection contains more than 71 million sequences, with a total size of 466 gigabases (Gb). Using such a framework to test the probes for nonspecific hybridization is overly time-consuming. To limit the selection process, it is advisable to reduce the volume of nonspecific sequences by including only those sequences that can hypothetically be present in the tested samples.

**Figure 1:**
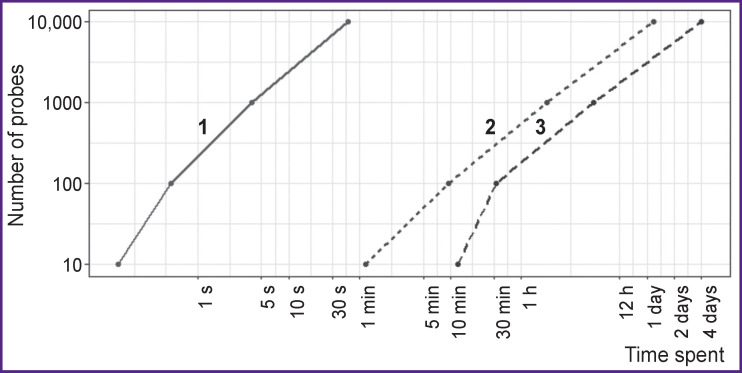
The sequence alignment using the BLAST algorithm: duration of the procedure plotted against the number of probes and the local base size A specified number of probes designed to detect the sequences of *C. pneumoniae* was aligned with the contents of bases of different sizes: *1* — base of target sequences (0.02 Gb); *2* — base of nonspecific sequences (165 Gb); *3* — base of nonspecific sequences (466 Gb). The time spent on the alignment procedure without processing the results is shown. The logarithmic scale of the axes is used

Using the literature, we have identified more than 40 taxa of microorganisms representing the relevant CAP pathogens and the biological material used for making the diagnosis (sputum, smears from the nasopharynx, oropharynx, etc.). A complete list of the taxa is presented in Appendix 1. For each taxon (using the disprose program), all associated sequences were downloaded from the NCBI Nucleotide collection database; those sequences formed the nonspecific database to be used further (8.7 million sequences with a total size of 165 Gb). Reducing the nonspecific database size made it possible to reduce around 5-fold the time spent later for testing the probe specificity (Figure 1).

#### Creating the candidate probe pool.

The pool of candidate probes was formed by virtual slicing a user-selected “parent” sequence into segments of a specified length. The sequence for slicing is user-defined (a frequent choice is the pathogen reference genome) and can be presented as a FASTA file obtained from any source or downloaded directly from the NCBI bank.

#### Testing the physical and chemical properties of the probes.

Since all probes in a DNA microarray must interact with target sequences under the same conditions (for example, at the same hybridization temperature), an important step is to determine the physicochemical properties of candidate probes. The disprose program provides the options for testing four physicochemical parameters allowing to control the conditions of hybridization, stability of the probe secondary structure: percentage of guanine and cytosine nucleotides (GC), the number of homo-repeats, melting temperature (Tm) estimate, and MFE (see the Table). In addition, the program provides the ability to change the computation parameters.

**Table:** Relevant physicochemical parameters of the probes

Parameter	Impact on probe performance	Values (by default)	References
Probe size	As the length of the probe increases, its discrimination potential decreases, but the efficiency and the hybridization signal increase	24–32 nb	[1, 12, 13]
GC content	Impact on the melting point: a low GC content reduces the hybridization efficiency; a high GC content increases the likelihood of nonspecific hybridization	40–60%	[2, 14–17]
Number of homo-repeats (identical nucleotides repeated in a row)	More than four identical nucleotides in a row increase the likelihood of nonspecific binding	<5 nb	[18]
Minimal folding energy (MFE)	The lower the MFE value, the higher the likelihood of secondary structure stabilization by the probe, leading to a decrease in its sensitivity and the efficiency of hybridization	≥–3 kcal/mol	[7, 8, 13, 19]
Melting temperature	The main condition of the hybridization reaction determines the buffer solutions characteristics. The hybridization temperature is about 5° below the melting point	55–60°C	[10, 13, 16]

#### Testing the probe specificity.

The algorithm implies a two-step specificity testing procedure. At the first step, the ability of a probe to hybridize to the target sequence is assessed by aligning the probe with the previously selected pathogen sequences using the BLAST algorithm. The results are then processed using specialized functions of the disprose package. During processing, for each probe, the number of target sequences, with which it got aligned under the required conditions (minimal alignment length, percentage of coverage, score, and E-value) is determined.

At the second step, the probes are tested for the specificity by aligning them with the sequences from the nonspecific pool.

As a result, for each probe, a list of target and nonspecific sequences, with which the probe can potentially interact is obtained. By analyzing both the number and nature of the specific and nonspecific interactions (with the help of the disprose functions), it becomes possible to select probes with specificity that is controlled *in silico*.

#### The final stage of the analysis.

In the event that the selected probes are not specific for all target sequences, the analysis cycle is restarted. In this case, the sequences uncovered in the first cycle make a new target bank of a smaller size; again, a new pool of candidate probes is created based on a new “parent” sequence. The cycles are repeated until probes able to hybridize with the given target sequences of the pathogen are found.

Thus, the proposed algorithm for the selection of discriminating probes includes three main stages, performed sequentially:

1. composing the list of target and nonspecific sequences, creating the local banks of sequences;
2. generating a pool of candidate probes, checking their physicochemical parameters;
3. testing the ability of candidate probes to hybridize with target and nonspecific sequences.

A list of disprose program functions, providing the implementation of this algorithm, and their brief characteristics are presented in Appendix 2.

### Additional features of the algorithm and its optimization

In addition to the main algorithm for the selection of specific probes, the disprose program implements additional functions: adding nucleotide adapters to probe sequences and annotating pathogen genome regions interacting with the probes. The possibility of using sequences from the whole genome sequencing projects — WGS (whole genome shotgun) is also available. Optimization of the algorithm performance for its successful implementation is among these additional features.

#### Working with WGS projects.

WGS projects are incomplete assemblies of genomes or chromosomes of prokaryotes and eukaryotes; these sequences are termed “contigs”. Including such contigs in the list of target sequences is problematic since each contig is considered an independent unit when aligned by the BLAST algorithm. Therefore, it would necessitate selecting a specific probe for each contig, giving rise to an excessive number of probes and increasing the time of the operation.

The proposed algorithm can be adjusted to WGS projects sequences through specialized functions of the disprose program, which allow considering all contigs of one genome as a single virtual sequence. In this case, probes that match one of the contigs are regarded as specific for the whole genome of the WGS project.

#### Optimizing the algorithm performance.

As the selection of specific probes involves the screening of several millions of candidate sequences, it is of paramount importance to develop a high-performance algorithm. The disprose program utilizes standard techniques for increasing the algorithms’ performance, such as storing intermediate data in the SQL database and running most of the functions in parallel (applicable for a multi-core server configuration). However, the main factor that determines the pace of computation is the order in which the program functions are applied.

**Figure 2:**
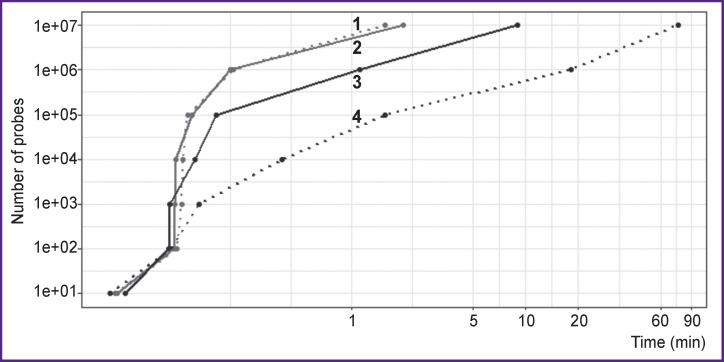
The time spent plotted against the number of probes and the type of computation The physicochemical parameters of probes for the detection of *C. pneumoniae* sequences were computed: *1* — test for the presence of homo-repeats; *2* — calculation of the GC percentage; *3* — calculation of the minimal folding energy; *4* — calculation of the melting point. The logarithmic scale of the axes is used

**Figure 3:**
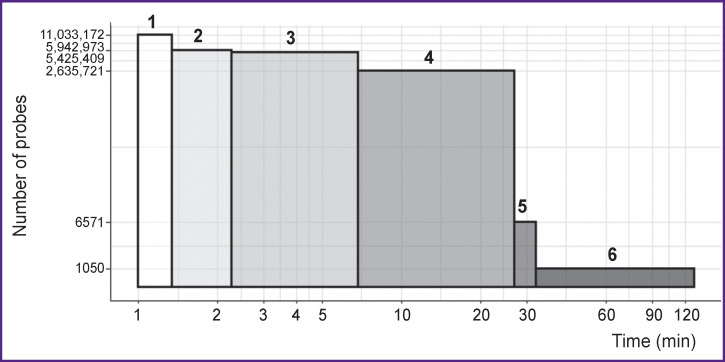
Time spent on the selection of probes for detecting *C. pneumoniae* sequences The stages of computation are shown: *1* — calculation of the GC percentage; *2* — checking for the presence of nucleotide homo-repeats; *3* — calculating the minimal folding energy; *4* — calculating the melting point; *5* — checking the hybridization, aligning the probe sequences to the target sequence base; *6* — verifying the specificity, aligning the probe sequences to the nonspecific sequence base. When performing the procedures, the number of probes gradually decreased due to the elimination of probes with unacceptable characteristics. For alignment procedures, the times spent on the procedure itself and on the data processing are indicated. The logarithmic scale of the axes is used

As shown in Figures 2 and 3, different operations of this algorithm differ in their performance, with the maximum time spent on aligning the probes with sequences from local banks when testing the specificity. That is why we recommend testing the probe specificity at the very last stage when most of the probes have already been eliminated from the candidate list.

The procedures for determining the physicochemical parameters of the probes are also relatively slow. Each indicator is calculated at a different speed (see Figure 2). The stages of calculating the percentage of GC and homo-repeats seem to be the most productive and allow one to immediately exclude probes with knowingly unacceptable characteristics. These steps must be executed first. Thus, when selecting probes for the detection of *C. pneumonia,* we used sequential elimination of candidate probes that did not meet the GC presence criteria, the number of homo-repeats, and the MFE levels. This approach allowed us to reduce the number of candidate probes from 11.0 to 2.6 mln before arriving at the stage of calculating the T_m_ — the slowest operation of the entire process. This reduction saved us the time for calculating the T_m_ by 3.7-fold (20.1 instead of 75.2 min).

#### Search for specific probes capable of detecting Chlamydophila pneumoniae.

We used the proposed algorithm and the disprose program to search for specific probes that allow the detection of *C. pneumoniae*. To create the list of specific sequences, metadata on the sequences available under the search query “Chlamydia pneumoniae * [organism] OR Chlamydophila pneumoniae * [organism]” were downloaded from the NCBI Nucleotide collection database. The resulting metadata set contained a total of 9062 records. According to further analysis, 17 whole genome sequences and 165 sequences of the WGS project were selected as targets. The target base had a size of 0.02 Gb. The list of target sequences is presented in Appendix 3. The nonspecific sequence base was made up of previously selected sequences associated with human genetic material, representatives of its normal flora and microbiota.

The sequence “Chlamydia pneumoniae TW-183, complete sequence” (ID number NC_005043 NCBI RefSeq) was chosen as the “parent” sequence to create the candidate probe pool. The parent sequence was sliced into segments of all possible lengths from 24 to 32 nucleotide bases (nb), which constituted the pool of candidate probes (11,033,172 probes).

When testing the physicochemical properties, we used the following selection criteria: the content of G and C in the range of 40–60%, the absence of homo-repeats of 5 nb and longer, and the MEF — not less than 0 kcal/mol. In the end, the T_m_ value of the probes was calculated. Most probes had T_m_ values close to 57°C. Due to that, we were able to accelerate the selection process by reducing the number of probes while keeping only those with T_m_ within 56.97–57.03°C. Thus, the total number of candidate probes selected in such way for the next stage was 6571.

To test the ability of candidate probes to hybridize specifically with target sequences, the probes were aligned with the either target or nonspecific sequences using the BLAST algorithm. To obtain the most specific probes, we established the following conditions for efficient hybridization: for hybridization with the target sequences, the identity should be 100% in the absence of point mismatches and nucleotide gaps; for hybridization with nonspecific sequences, the identity is required to be 50% or higher.

Of the candidate pool, 6380 probes effectively interacted with all target sequences *in silico.* To shorten the time required for detecting possible nonspecific hybridizations, the number of candidate probes was reduced to 1050 by narrowing the range of acceptable T_m_ values to 56.994–57.006°C. Probes that efficiently hybridized with at least one nonspecific sequence were excluded from the final pool.

The above operations of the algorithm resulted in 100 specific discriminating probes, which provided the differential detection of *C. pneumoniae* among other pathogens. Due to their high specificity, the selected probes can be used as a functional basis for DNA microarrays designed to identify actual causative agents of CAP. The total time for selecting the probes using the disprose program was 130 min. A list of selected probes and their characteristics is presented in Appendix 4.

## Discussion

The proposed algorithm for selecting probes for a DNA microarray involves the identification of target sequences. The specificity and discriminatory potential of the probes (i.e. the ability of a DNA microarray to detect a specific pathogen) directly depend on the conservatism of the target sequence.

Probes for highly conserved regions of the genome, such as the *16S* and *23S* rRNA genes, although less specific, allow one to distinguish between bacteria belonging to different species. Probes for less conservative sequences, for example, bacterial genes *recA*, *gyrB*, *rpoB*, are able to distinguish between strains of bacteria within the same species [1, 12].

If there is no information on the degree of conservatism in the given genome, it is possible to search for these data using a set of target sequences and the method of their multiple alignment. However, multiple alignment of a large number of long sequences (for example, genomes) requires significant computing power and takes a long time [20].

In the disprose program, a different approach to searching for target regions is implemented; that is to generate a large number of short probes and align them into a set of sequences using the BLAST algorithm. This process is less demanding in terms of equipment, it is well implemented using a parallel mode, and it is ten times less time-consuming. Probes aligned to the full target sequence list with 100% coverage are considered to be specific to conserved regions of the sequence pool. Thus, varying the list of target sequences using the disprose program, makes it possible to select probes for designing DNA microarrays with different discriminatory potential.

We tested the developed algorithm by searching for specific probes that would allow for differential detection of *C. pneumoniae* in clinical samples. *C. pneumoniae* is one of the many microorganisms that cause CAP. The cumulative share of this and other “atypical” causative agents of CAP vary from 8 to 30% of cases [21, 22], and their timely detection can accelerate and improve the diagnostic process [23]. This application of the disprose program — the search for probes capable of detecting *C. pneumoniae* — demonstrated the potential of the developed algorithm.

As a result of the algorithm operation, one hundred probes with high specificity for the target pathogen were selected from the large-size pool of candidate probes. After comparing between the sites of origin of the probes and fragments of the annotated reference genome, we found that most of the probes originated from the genes that encoded for enzymes, chaperone proteins, and also regulators of the cell cycle (see Appendix 4). These genes contain regions highly conserved for *C. pneumoniae* and may be of interest not only for the diagnostic purpose but also for phylogenetic studies.

## Conclusion

An algorithm has been developed to search for specific probes able to identify human pathogens of bacterial and viral origin. The algorithm is implemented as the disprose computer program written in the R language; its performance has been demonstrated by identifying the probes for detecting *C. pneumoniae*. The algorithm and program for the selection of probes have a number of advantages:

universality — the algorithm is aimed at finding specific areas in a set of sequences of any size and can be easily adapted to solve a wide range of tasks;

modularity — the execution of the algorithm occurs in several stages, their order is determined by the user, while any stage can be skipped or performed using a third-party software product;

openness — the initial code of the disprose package is publicly available and can be modified in accordance with the task;

usability — the algorithm can work with popular databases of genetic information (NCBI, GISAID) and local databases.

The flexibility and openness of the program provide for expansion of the scope of its application.
